# OK-432 Treatment of Ranula Intruding into the Cervical Region

**DOI:** 10.3390/clinpract12020025

**Published:** 2022-03-28

**Authors:** Nobuo Ohta, Shion Shirane, Shigeru Fukase, Rei Kawata, Teruyuki Sato, Nozomi Satani, Takahiro Suzuki

**Affiliations:** 1 Division of Otolaryngology, Tohoku Medical and Pharmaceutical University Hospital, 1-12-1, Fukumuro, Miyaginoku, Sendai 983-8512, Japan; shirane_shion@yahoo.co.jp (S.S.); rei.kawata@hosp.tohoku-mpu.ac.jp (R.K.); teruyukisato@gmail.com (T.S.); pilsaper@yahoo.co.jp (T.S.); 2 Fukase Clinic, 2-7-30, Nanokamachi, Yamagata 990-0042, Japan; ads19557@nifty.com; 3 Division of Radiology, Tohoku Medical and Pharmaceutical University Hospital, 1-12-1, Fukumuro, Miyaginoku, Sendai 981-8512, Japan; nozomisatani@tohoku-mpu.ac.jp

**Keywords:** OK-432, ranula, anterior cervical region

## Abstract

Objectives: Plunging ranula intruding into the cervical region is rare and a standard therapy has not yet been consolidated. This paper investigates the outcomes and side effects of OK-432 treatment in patients with a ranula extending into the cervical region. Methods: The study design and setting consisted of a planned data collection at Tohoku Medical and Pharmaceutical University and Fukase Clinic. Eight patients with ranula extending into the cervical region received OK-432 treatment between January 2016 and February 2019. OK-432 treatment was performed for patients with ranula extending into the cervical region. Results: In all patients, a total shrinkage and marked reduction in lesions were observed without local scars or deformations after OK-432 treatment. Complications were local swelling and mild fever (37.5–38.5 °C), which lasted a few days in half of the patients. Conclusions: OK-432 treatment is straightforward, secure, and efficacious and can be substituted for surgery in the treatment of ranula extending into the cervical region.

## 1. Introduction

Ranula is a commonly encountered disorder for general otolaryngologists, but plunging ranula intruding into the cervical region is rare [1]. Plunging ranula appear as pools of mucous are surrounded by fibrous tissue and chronic inflammatory cells, including histiocytes. Epidermoid cysts, thyroglossal duct cysts, cystic hygroma and branchial cleft cysts should be kept in the mind as differential diagnoses [2,3]. Trans-oral excision of the sublingual gland and the evacuation of the ranula are used as standard treatments [2,3]. However, potential side effects, such as insufficient surgery, cosmetic problems, nerve injury, and vascular damage, can occur [1,2,3]. Recently, OK-432 treatment has been reported as efficacious in the treatment of ranulas [4,5,6,7,8,9]. However, its effectiveness in patients with ranula intruding into the cervical region is not clear. To clarify this point, we examined it in this paper.

## 2. Materials and Methods

Patients: Eight patients with ranulas intruding into the cervical region were treated with OK-432 at Tohoku Medical and Pharmaceutical University and Fukase Clinic between January 2016 and February 2019. One representative case is presented here. Essentially, OK-432 treatment was performed on an outpatient basis without hospitalization. Informed consent was obtained under protocols approved by the Institutional Review Board (2016-2-020). The diagnosis of ranula was confirmed by clinical symptoms, MRI or CT, and aspiration cytological examination. The presence of peripheral fibrosis, lined with a non-keratinizing stratified squamous epithelial layer with a central pool of mucin, inflammatory cells and mucinophages was in accordance with ranula.

OK-432 therapy: OK-432 seems to be safer and more effective than other sclerosing agents, such as boiling water, hypertonic saline, ethanol, tetracycline, cyclophosphamide, sodium morrhuate, and bleomycin [6,7]. The fluid component of each ranula was completely suctioned, with compression as needed, with an 18- or 27-gauge needle. The suctioned fluid was examined in the laboratory. The OK-432 (Picibanil, Chugai Pharmaceutical Co., Tokyo, Japan) was diluted with saline solution (1 to 2 Klinische Einheit (KE) per mL; 0.1 to 0.2 mg/mL) and injected into the ranula with a new syringe. Successful injection was performed without resistance. Patients received a second or third treatment if the response to the previous was insufficient.

Follow-up: Regular examination was performed for all patients for a mean of 25.3 months (range, 12–42 months) after the last treatment. Prophylactical analgesics were prescribed for possible fever. Induration and redness of the skin at the injected site were observed the day after injection. Local and systemic conditions were examined, and all patients were evaluated at weeks 1 and 6 after OK-432 treatment. We defined “total shrinkage” of cysts as complete absence, “marked reduction” as a reduction of more than 50%, and “partial reduction” as a reduction of less than 50%, clinically or by using computed tomography or magnetic resonance imaging.

## 3. Results

**Figure 1:**
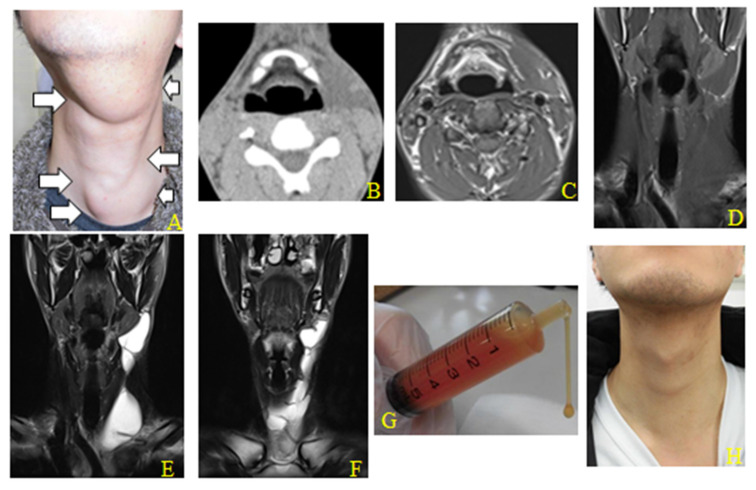
Totally shriveled right cervical lesion in a 22-year-old Japanese man after single treatment with OK-432. (**A**) Local findings before OK-432 treatment, showing the ranula intruding into the left anterior cervical region (16.4 cm) (arrow indicating the diffuse swelling of the anterior cervical region). (**B**)Initial axial computed tomography before OK-432 treatment showing the ranula intruding into the anterior cervical region. (**C**,**D**) Initial axial and coronal T1-weighed magnetic resonance image before OK-432 treatment, showing the ranula intruding into the anterior cervical region. (**E**,**F**) Initial coronal T2-weighted magnetic resonance image before OK-432 treatment, showing the ranula diving into the submandibular region. (**G**) The lesion puncture fluid was very sticky mucus. (**H**) Local findings 6 weeks after OK-432 treatment showing totally shriveled lesion.

OK-432 treatment was performed for eight patients with ranula intruding into the cervical region (Table 1). The mean age of patients was 25.8 years (range, 4–65 years). The maximum size of the ranula ranged from 7.7 to 16.4 cm (mean, 11.9 cm). The mean number of OK-432 treatments was 1.4 (range, 1–3), and the mean observation period was 25.3 months (range, 12–42 months). The mean treatment dose of OK-432 was 1.9 KE (range, 1–2 KE), and the mean total dose was 2.8 KE (range, 1–7 KE). The clinical results of the OK-432 treatment did not to depend on the cyst size, location or the patient’s age. Ten OK-432 treatments were performed on eight lesions, but six of the eight patients required only one treatment. All cases showed a total shrinkage or marked reduction (observation for more than 1 year after the final injection without any recurrence or further treatment) after one to three injections. A representative case is shown in Figure 1. This case showed total shrinkage after a single OK-432 treatment.

No patients had any serious complications apart from local swelling of the injected site and moderate-grade fever (37.5–38.5 °C) for a few days after injection, usually manageable by antipyretics. Local scar or deformation after OK-432 treatment was not observed in any patients.

## 4. Discussion

Ranula is common disorder of the oral cavity [1]; however plunging ranula intruding into the cervical region is rare [2]. The reported conservative and surgical procedures for ranula include a simple aspiration, fenestration, incision and drainage, excision, and excision along with the sublingual gland [1,2,3,8]. Potential surgical complications that need to be considered include vascular damage, nerve injury, recurrence due to insufficient surgery, and cosmetic problems [1,2,3,4]. OK-432 is a lyophilized streptococcal preparation made from the Su strain of A-group *Streptococcus pyogenes* incubated with penicillin. It was developed as an immunotherapeutic agent of cancer. We reported previously that OK-432 therapy is effective in the treatment of various cystic diseases, including ranula, lymphangioma, salivary mucocele, auricular hematoma and branchial cleft cyst. OK-432 seems to be more safe and effective than other sclerosing agents, such as boiling water, hypertonic saline, ethanol, tetracycline, cyclophosphamide, sodium morrhuate, and bleomycin. Although the complication rates with treatment by these sclerosing agents are minimal, the limited success and unpredictable local scarring, as well as systemic side effects caused by spread of the agents beyond the endothelial lining of the lesion, have been observed. Bleomycin, in particular, can cause serious side effects, including fibrosis of the lung, independent of the total dosage [10,11,12,13,14,15,16].

In the treatment of plunging ranula, there was no significant difference in cure rate between the intraoral and transcervical approaches, although they both showed higher cure rates than OK-432 treatment [8]. The advantages of OK-432 treatment over surgical modalities can be summarized as follows: (1) special equipment and hospitalization were not required for OK-432 treatment; (2) local scar or deformation was not observed after OK-432 treatment; and (3) the time for procedure is short [6,10,11]. OK-432 treatment is cosmetically and economically more beneficial than surgical modalities and can be used as a substitute for surgery [7,10,14].

## 5. Conclusions

OK-432 treatment is straightforward, secure, and efficacious and can be substituted for surgery in the treatment of ranula extending into the cervical region.

## Figures and Tables

**Table 1:** Clinical Characteristics.

Case	Age	Sex	Size	No of Tx	Dose (KE)	Total Dose (KE)	Follow-Up	Outcome	Further Tx	History
1	22	F	16.4	1	2	2	12	TS	none	none
2	9	M	9.4	1	2	2	15	TS	none	none
3	5	F	8.1	1	2	2	36	TS	none	none
4	65	M	15.4	1	2	2	12	TS	none	HT, af, CI
5	11	M	11.2	3	2	7	21	TS	none	none
6	4	M	7.7	1	1	1	28	Ts	none	none
7	51	F	14.8	1	2	2	36	TS	none	HT, CI
8	39	F	12.1	2	2	5	42	MR	none	none

Size, maximum diameter (cm); TS, total shrinkage; MR, marked reduction; Follow-up is in months; No., number; Tx, treatments; Further Tx, Further treatment; Dose is in KE (Klinische Einheit); Total dose is in KE; HT, hypertension; af, atrial fibrillation; CI, cerebral infarction.
